# What is the Role of Over 100 Excipients in Over the Counter (OTC) Cough Medicines?

**DOI:** 10.1007/s00408-020-00390-x

**Published:** 2020-09-05

**Authors:** Ronald Eccles

**Affiliations:** Cardiff School of Biosciences, Cardiff University, Museum Avenue, Cardiff, CF10 3AX UK

**Keywords:** Cough, Excipients, Color, Thickeners, Flavors, Sweeteners, Menthol, Placebo effect

## Abstract

Most medicines are white bitter powders that are formulated as tablets and capsules but cough medicines are an exception where the taste and appearance of the medicine are more important to the patient than the pharmacology of the active ingredient. Excipients are generally defined as any ingredient in a medicine other than the active ingredient. In most medicines excipients play a supportive role in delivering the medicine, but in the case of cough medicines, excipients have more important and complex roles and they can also be the main active ingredient of the cough medicine as menthol, glycerol, and sugars, which are declared as active ingredients. This review searched the United Kingdom electronic medicines compendium (emc) and found over 100 excipients in 60 different liquid formulations of over the counter cough medicines. The excipients were divided into functional groups: sweeteners, thickeners, flavors, colors, antimicrobials, and buffers, and the incidence and function of the different excipients is discussed. When considering the efficacy of a cough medicine, clinicians and pharmacists tend to think of the pharmacology of antitussives such as dextromethorphan or expectorants such as guaifenesin, and they rarely consider the role of excipients in the efficacy of the medicine. This review discusses the functions and importance of excipients in cough medicines and provides some new information for clinicians, pharmacists, and all interested in the treatment of cough when considering the composition and efficacy of a cough medicine.

## Introduction

Excipients are generally defined as any ingredient in a medicine other than the active ingredient. Their role in most medicines is to act as a stable vehicle for the delivery of the active ingredient in the form of a tablet, capsule, cream, or liquid. In most medicines, the excipients play a supportive role in delivering the medicine, but in the case of cough medicines, excipients have more important and complex roles and they can also be the main active ingredient of the cough medicine. Cough medicines are unique in that the most common form of the medicine is a viscous sweet syrup [1]. The active ingredients such as the antitussive dextromethorphan or expectorant guaifenesin, etc. could be delivered to the patient as tablets or capsules without any loss of pharmacological activity, but because of the history of cough medicines being formulated from food substances such as honey, a sweet viscous syrup formulation is expected by the patient consumer [2]. Although cough medicines are controlled by medical regulations, they have many similarities to foods and beverages, and most of the excipients now found in cough medicines have been derived from the food and beverage industry. There is a great advantage in using food and beverage additives as excipients in cough medicines as they are generally recognized as safe (GRAS) by regulatory authorities and this makes the registration of new formulations of cough medicines much easier for companies marketing cough medicines. This paper will discuss the role of excipients in cough medicines. The paper will discuss excipients in liquid formulations of cough medicines and will not discuss tablet, lozenge, and capsule formulations. The OTC cough medicines discussed in this article are for the treatment of acute cough associated with acute upper respiratory tract infections, but they may also be used by patients suffering from chronic cough but no information on this use has been found in the literature.

**Fig. 1:**
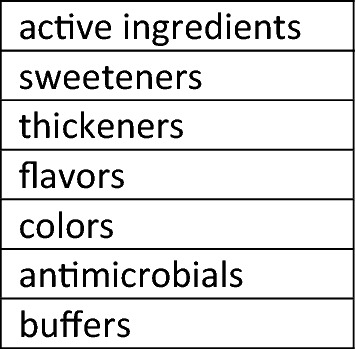
Functional components of a cough syrup medicine

The composition of a cough medicine can be considered as made up of seven functional components as illustrated in Fig. 1. The declared active ingredients are usually pharmacologically active compounds such as dextromethorphan, guaifenesin, etc. but can also include excipients such as menthol, glycerol, and sugars. Sweeteners, thickeners, flavors, colors, antimicrobials, and buffers are excipients and will be discussed below as functional components of a cough medicine. “Other” excipients that did not fit into the functional categories are also discussed.

## Source of Data

The information on cough medicines has been taken from the electronic medicines compendium (emc) [3] which contains freely available information about medicines licensed for use in the United Kingdom and lists over 14,000 documents. The search term cough was used on the database in June 2020 and this generated data on 105 medicines used to treat cough. Medicines in tablet, capsule, or pastille form, multi-ingredient medicines, and medicines only available on prescription were excluded from the list, and this left 60 cough medicines in liquid form. These cough medicines were freely available on general sale or available at pharmacists. The list of excipients in the summary of product characteristics (SmPC) for each of the 60 cough medicines was searched and a list of all excipients and their frequency of occurrence was made. 109 excipients were listed and their properties were mainly determined by reference to the Handbook of Pharmaceutical Excipients [4] or other sources as referenced.

## Sweeteners

Sweeteners are common excipients in cough medicines. As discussed above, natural honey was the first cough medicine and is still popular as a cough treatment today [5]. Sweeteners and thickeners mimic the sweetness and viscosity of natural honey and continue the tradition of cough medicines being formulated as sweet viscous syrups. A list of sweeteners and the number of medicines containing each sweetener is shown in Table 1. Natural sugars such as liquid sugar, liquid glucose, and sucrose were the most common sweetener excipients. Natural sugars such as glucose and sucrose were also declared as active ingredients in 10 medicines and it is stated that they “have demulcent properties and will soothe irritated sore throats and possibly block sensory cough receptors within the respiratory tract.”

**Table 1:** List of sweeteners, with frequency of each sweetener from 60 cough medicines

Sweeteners	Frequency
Liquid glucose	30
Sucrose	30
Saccharin sodium	23
Liquid sugar	20
Sorbitol	13
Maltitol liquid (E965)	6
Acesulfame K	5
Sodium Cyclamate	5
Natural sweetness enhancer	3
Ammonium glcyrrhizate	2
Partial Inverted Syrup (sucrose, fructose, and glucose)	1
Sucralose	1
Xylitol (E967)	1

Artificial sweeteners such as sucralose, acesulfame K, sodium cyclamate, xylitol, and saccharin sodium can provide a low calorie cough medicine or be used to increase the sweetness of a medicine containing natural sugars. Sorbitol is a natural sugar but can also be synthesized from glucose; it provides sweetness with fewer calories than natural sugars such as glucose. Malitol can be synthesized from the natural sugar maltose and has the advantage over Sorbitol in that it is not metabolized by oral bacteria and therefore does not contribute to tooth decay. Malitol can be used as a low calorie sweetener and it has the advantage that it does not crystallize easily and therefore is less likely than natural sugars to cause bottle tops to stick.

Ammonium glychyrrhizate (glycyrrhizin) is a triterpene glycoside found in the roots of licorice plants (glycyrrhiza glabra). The latin name is derived from the Greek word ‘glykos’ meaning sweet and it has been used as a sweetener and medicine for thousands of years in many Asian countries [6, 7]. “Glycyrrhizin has a sweet taste with a characteristic licorice taste sometimes described as “cooling.” The sweetening potency is about 50 times that of sucrose. The sweetness is slow in onset and tends to linger” [8]. Glycyrrhizin does not provide any calories and therefore can be used to greatly enhance the sweetening contributed by natural sugars and provide a licorice taste.

### Role of Sweeteners in Cough Medicines

Sweeteners not only improve the taste of cough medicines and make them more pleasant to take but they also reduce the perception of the bitter taste of active antitussives such as dextromethorphan [9]. A review on the role of sweeteners in cough medicines has stated that most cough medicines are formulated as sweet syrups and that the sweet taste provides the major benefit of the medicine and is more important than the pharmacologically active ingredient in the medicines [1]. The review proposed that sweeteners may act as antitussives in two ways, firstly by stimulating salivation and airway secretions that soothe and lubricate the inflamed pharynx and secondly by the generation of endogenous opioids in the brainstem area that controls cough. This proposal was tested in a study that found that the sweet taste of sucrose increased cough reflex thresholds [10, 11]. Although it is stated in the SmPC of 10 medicines that natural sugars can “possibly block sensory cough receptors within the respiratory tract,” no evidence has been found in the literature to support this mode of action.

## Thickeners

Nearly all the cough medicines contained a thickening excipient and the list of thickeners and the number of medicines containing each thickener is shown in Table 2.

**Table 2:** List of thickeners, with frequency of each thickener from 60 cough medicines

Thickeners	Frequency
Glycerol	48
Propylene glycol	20
Hydroxyethylcellulose	14
Carmellose sodium	6
Arrowroot	3
Xanthan gum	3
Acacia (E414)	2
Maltodextrin	2
Povidone	2
Carrageenan	1

The most common thickening agent used in cough syrups was glycerol also known as glycerine and this was found in 48 of the 60 products. Glycerol was also listed as an active ingredient in 17 cough medicines and the SmPC often stated that “Glycerol has demulcent properties and may possibly block sensory cough receptors in the respiratory tract.” No evidence has been found in the literature to support glycerol acting on sensory cough receptors. Glycerol is popular as an excipient in cough medicines because it serves multiple functions; acting as a sweetening agent (0.6 times the sweetness of sucrose), a solvent, lubricant, antimicrobial, humectant, and preservative. The special properties of glycerol as an ingredient of cough medicines have been recently reviewed and the article concludes that “a simple linctus containing glycerol with flavorings such as honey and lemon is a safe and effective treatment for cough in children and adults” [12]. Glycerol is a small molecule with three carbon atoms and its viscous nature is because each of the carbon atoms has a hydroxyl group attached and these hydroxyl groups can bind to other hydroxyl groups by hydrogen binding with water, which makes glycerol very soluble in water, or by hydrogen binding to other glycerol molecules, which causes aggregation of glycerol molecules and means that glycerol does not flow as well as water and is rather viscous [13].

Propylene glycol was the second most commonly used thickening agent found in 20 of the cough medicines. Propylene glycol has a three carbon chain with two hydroxyl groups and has similar properties to glycerol as a thickening agent. Its solubility in water and viscosity are due to the hydrogen binding of the two hydroxyl groups on the molecule. It has a sweet taste and has useful properties as a solvent, antimicrobial, preservative, humectant, lubricant, and demulcent.

Glycerol and propylene glycol are unusual as a thickening agent as most thickening agents are synthetic long-chain polymer molecules such as hydroxyethylcellulose (Hyetellose, Natrosol), polyvinylpyrrolidone (Povidone), carboxymethylcellulose (Carmellose), and maltodextrin. These synthetic thickening agents are viscous because of the long molecular chains and they are without any taste and are not absorbed or metabolized and therefore safe. In addition to the synthetic thickening agents, a range of natural products are used as thickening agents because they are long-chain sugars or cellulose. Carrageenan is obtained from various red sea weeds. Acacia (Arabic gum) is obtained from trees (Acacia Senegal) in the Sudan region. Arrowroot is a starch substance obtained from the roots of several tropical plants. Xanthan gum is obtained by fermenting carbohydrates with the bacterium Xanthomonas campestris.

The synthetic thickening agents are by far the most commonly used agents in cough medicines and this is because they are easily standardized, and the industrial source is stable. Natural thickening agents are more difficult to standardize and source and they may have a place in medicines that claim to be herbal or natural products.

### Role of Thickeners in Cough Medicines

Most cough medicines are formulated as thick viscous syrups and this may be due to consumers considering a thick syrup as having stronger effects than a watery liquid. The viscous formulation may also be historical as viscous honey was one of the first cough medicines, used over 100 of years, and still popular today as an ingredient in cough medicines [5, 14].

Thickeners may also enhance the sensory impact of a cough medicine by prolonging the residence of the medicine in the mouth and therefore prolonging the duration of any sweet taste of the medicine. Some of the thickeners, such as glycerol, also have demulcent and lubricating properties that will help to soothe an inflamed pharynx [12].

Thickeners such as glycerol also provide a sweet taste, and others such as carrageenan can support antiviral and antibacterial claims [15]

## Flavors

Fifty-one different flavors were found in the search on 60 cough medicines. Some of these fell into single flavor groups such as raspberry flavor, which consisted of four different excipients (raspberry flavor in 8 medicines, raspberry flavor 503.850/T in 2 medicines, raspberry flavor F2126 in one medicine, and raspberry juice concentrate in one medicine). This gave a total of 12 references to raspberry flavor but since one medicine had 2 raspberry flavor excipients, the total number of medicines containing a raspberry flavor excipient was 11 medicines as illustrated in Table 3. Similarly, in Table 3, aniseed flavor was a combination of 3 excipients, anise oil in 14 medicines, aniseed flavor in 6 medicines, and aniseed flavor 545008E in 2 medicines.

**Table 3:** List of flavors, with frequency of each flavor from 60 cough medicines

Flavor	Frequency
Menthol, levomenthol	34
Anise oil, Aniseed flavor	22
Caramel	19
Capsicum tincture	13
Raspberry flavor	11
Cough syrup 513277	9
Peppermint oil	9
Honey and flavors	9
Blackcurrant flavor and juice	9
Black treacle	8
Lemon oil, juice, essence, flavor	7
Licorice Extract and flavor	6
Condensed milk flavor F12516	5
Apple flavoring 5112OIE	4
Cough mixture flavor	4
Pumilio Pine Oil	4
Blackberry flavors	4
Food flavor 511630E	3
Strawberry flavor	2
Hot mix flavor 538842T	1
Custard Flavor	1
Bitterness blocking flavor 84E260	1
Cooling flavor 539692T	1
Cherry/Grenadine flavor	1
Distilled lime oil	1
Flavored compound FC 900853	1
Grenadine flavor 514485E	1
Imitation peach flavor	1
Raspberry aroma flavoring	1
Strong Ginger Tincture	1
Tingling flavor 538723T	1
Vanillin	1

By grouping similar flavors together Table 3 condenses 50 excipients into 30 flavors. The most common flavor was menthol (levomenthol is commonly referred to as menthol), which was present in 34 of the 60 medicines, and in one medicine was combined with cooling flavor 539692T. One could also include peppermint oil (present in 9 medicines) as related to menthol, as this oil often contains up to 50% menthol [16].

Other common flavors were aniseed flavors, present in 22 medicines, caramel in 19 medicines, and capsicum and Hot mix flavor 538842T, which give a warm pepper flavor, with capsicum present in 13 medicines. Medicines formulated for children often contained a fruit flavor such as raspberry, blackcurrant, or blackberry, and honey flavor was also common in medicines for children.

Licorice extract and flavor was found in 6 medicines and this not only provided a distinct flavor but also enhanced the sweetness of the medicine as discussed under sweeteners.

The large number of flavors illustrates how cough medicines have transitioned into consumer products that more resemble foods or confectionery than medicines with flavors in cough medicines such as apple, strawberry, peach, cherry, custard, vanillin, and condensed milk all being common flavors in foods and confectionery.

The science of food flavoring has become more and more sophisticated with many new synthetic flavors and special molecules designed to enhance and modify flavors, and these synthetic flavors have found their way into use in cough medicines such as Tingling flavor 538723T, which enhances the oral sensations associated with the cough medicine, and Bitterness blocking flavor 84E260. Pharmacologically active antitussives such as dextromethorphan have a bitter taste that is unpalatable to children and most adults and this bitter taste can be masked by sweeteners but can also be blocked by excipients such as Bitterness blocking flavor 84E260. The masking of bitter taste by synthetic molecules is a well-developed field of food technology, and there is much research into this area because of its commercial significance [17]

### Role of Flavors in Cough Medicines

Flavors provide much more than just the taste of a cough medicine as they are often declared as the active ingredient. Menthol is declared as an active ingredient in 11 cough medicines and the supporting statement in the SmPC is that “Menthol has mild local anesthetic, decongestant and antitussive properties.” Menthol is often described as a nasal decongestant but it does not decongest but provides an increased sensation of nasal airflow due to stimulation of sensory receptors such as transient receptor potential melastatin (TRPM8) [18, 19]. Menthol is a common ingredient in cough medicines and there is some support for antitussive activity from studies on healthy volunteers where cough was induced by either citric acid or capsaicin inhalation [10, 20, 21].

Capsicum tincture was found in 13 medicines and declared as an active in one medicine as a traditional herbal medicine. Capsicum tincture is derived from the pepper plant and contains capsaicin which has a strong burning taste or pungency due to stimulation of sensory receptors such as transient receptor potential cation channel subfamily V member 1(TRPV1) [22]

Licorice extract is declared as an active in 4 cough medicines and capsicum in one medicine and their use as an active ingredient is supported in the SmPC by reference to their use as traditional herbal medicinal products.

Honey is declared as an active in one medicine and the SmPC states that honey acts as a demulcent and provides a soothing medium for an irritated throat.

Lemon flavor in combination with citric acid monohydrate are declared as actives in one medicine and the SmPC states that “the citric acid monohydrate and the lemon oil, both add to the sharpness of the product and enhance the flavor.”

The wide range of flavors indicates how the cough medicine market has diversified to suit different consumers. Adults who prefer a traditional flavor of medicine are likely to opt for flavors such as menthol, peppermint, capsicum, treacle, aniseed, and ginger, whereas children may opt for a fruit flavor such as raspberry, blackcurrant, blackberry, peach, or more familiar food flavor such as condensed milk, custard, and vanillin. Flavors such as honey and lemon may appeal to both adults and children.

Flavors add to the sensory impact of the cough medicine and this is important to enhance any placebo effect of the medicine. The placebo effect has been proposed to contribute up to 85% of the efficacy of some cough medicines [23], and the sweet or medicinal taste of the medicine with other sensory signals such as cooling, heat, and tingling sensations all contributing to maximize the sensory impact and the belief in the efficacy of the medicines.

## Colors

The range of color excipients found in the cough medicines is shown in Table 4. A caramel (yellow to red or brown color) was the most common color found in the cough medicines, and caramel flavor was also one of the most common flavors. Caramel color is one of the most common colors used in foods and drinks and it has a long history of use and is prepared by heat treatment of sugars. Yellow and red were also common colors for cough medicines with the deep red color of anthocyanin found in blackcurrant flavor medicines.

**Table 4:** List of colors, with frequency of each color from 60 cough medicines

Colors	Frequency
Caramel (E150) (brown)	9
Sunset Yellow (E110) (yellow)	9
Ponceau 4R (E124) (red)	7
Caramel T12 (brown)	5
Amaranth (E123) (dark red)	4
Anthocyanin (dark red, blackcurrant)	4
Quinoline yellow (yellow)	3
Carmoisine (E122) (red)	1
Patent Blue V (blue)	1

Caramel and anthocyanin colors are derived from natural plant sources but the other colors such as Sunset yellow, Ponceau 4R, Quinolone yellow, Carmoisine E122, Armaranth E123, and Patent blue V are synthetic colors. Synthetic colors have been discussed as possible causative agents in rare side effects to medicines such as urticaria but there has not been any move towards use of more natural color excipients as these are often unstable and their coloring power is lower than that of synthetic dyes [24].

The color of tablets has been shown to influence the perception of their properties as sedatives or stimulants with red yellow and orange associated with stimulant effects, and blue and green colors with tranquilizing effects [25]. The color of a cough medicine may influence perception about its efficacy and enhance its placebo effect, although no published research has been found on the effect of color on the placebo effect of cough medicines [26].

## Antimicrobials

Antimicrobials or preservatives are found in most foods and cough medicines as they prolong the shelf life of the food or medicine, especially cough medicines which are formulated as liquids and where the bottle containing the liquid may be opened several times a day and maybe kept for months at home for future use. A list of the antimicrobials found in the cough medicines is shown in Table 5.

**Table 5:** List of antimicrobials, with frequency of each antimicrobial from 60 cough medicines

Antimicrobials	Frequency
Benzoates	43
Ethanol 96%	23
Potassium sorbate	4
Sorbic acid	1
Isopropyl alcohol	1
Toluflavor solution	1

The most common antimicrobials found in cough medicines were benzoic acid derivatives found in 43 of the medicines, such as sodium benzoate, methyl hydroxybenzoate, methyl parahydroxybenzoate, sodium methyl hydroxybenzoate, sodium propyl hydroxybenzoate, and sodium ethyl parahydroxybenzoate. These benzoic acid derivatives have bacteriostatic, and fungistatic properties by interfering with the metabolism of glucose in microbes [27]. Ethanol 96% was found in 23 medicines and it acts not only as an antimicrobial but also as a solvent.

Potassium sorbate is a commonly used antifungal agent although it does have antibacterial activity. Its potency as an antimicrobial is dependent on its state of dissociation, and this is pH dependent with practically no antibacterial activity above pH 6. Sorbic acid is less stable and less soluble than potassium sorbate and has similar properties to potassium sorbate.

Isopropyl alcohol is used mainly as a solvent but it also has antimicrobial activity at concentrations greater than 70% v/v. Isopropyl alcohol is about twice as toxic as ethanol and is not widely used in cough medicines and was found in only one of the medicines found in the search.

Toluflavour solution is a mixture of; alcohol, benzoic acid, cinnamic acid, cinnamon oil, ethyl cinnamate, sucrose, vanillin, and water, and this mix of antimicrobial and flavors was found in only one cough medicine.

## Buffers

Buffers are important excipients in cough medicines as they maintain an optimal pH for the disassociation and solubility of active ingredients and other excipients. The most common buffer found in cough medicines was citric acid either as the monohydrate, anhydrous citric acid, or sodium citrate, and was found in nearly all the medicines. Theses excipients are listed functionally as an acidifying agents, antioxidants, buffering agents, chelating agents, and flavor enhancers (Table 6).

**Table 6:** List of buffers, with frequency of each buffer from 60 cough medicines

Buffers	Frequency
Citric acid monohydrate	49
Sodium citrate	23
Citric acid anhydrous	5
Acetic acid glacial	2

Glacial acetic acid was used as an acidifying agent and buffer in two medicines, and it is also reported to have antimicrobial activity and can be used as a preservative.

## Other Excipients

The chelating agent disodium edetate was found in two medicines and it has the property of binding and neutralizing heavy metal ions.

Chloroform was found as an excipient in one expectorant cough medicine. Chloroform has a long history of use in cough medicines but its toxicity has led to very little use in modern medicines [28].

Clove oil was found in one traditional herbal cough medicine and it has some local anesthetic properties [29].

Purified water was found in all cough medicines.

## Discussion

When considering the efficacy of a cough medicine, clinicians and pharmacists tend to think of the pharmacology of antitussives such as dextromethorphan or expectorants such as guaifenesin, and they rarely consider the role of excipients in the efficacy of the medicine. This short review highlights the functions and importance of excipients in cough medicines and provides some new information for clinicians, pharmacists, and all interested in the treatment of acute cough when considering the composition and efficacy of a cough medicine.

As mentioned in the introduction, cough medicines are unique among medicines in that they are usually formulated as sweet viscous syrups when the active ingredients such as antitussives and expectorants could easily and effectively be administered as a tablet or capsule. The formulation of cough medicines as syrups goes back to the first cough medicines formulated from honey products and then later as sugar-based products. The fact that the viscous sweet formulation has persisted for hundreds of years indicates that this formulation is providing some benefit to the patient with acute cough and that the advent of pharmacologically active agents such as dextromethorphan and so on meant that these drugs were merely added to the sweet syrup formulation which had already proved to be of benefit to patients.

Glycerol is a sweet viscous syrup that has to some extent replaced honey as the most common excipient in cough syrups. Glycerol was found in 48/60 medicines and was declared as the active ingredient in 17 medicines. One product, which declared glycerol as the active ingredient to treat cough, contained 100% glycerol with no other excipient present in the formulation. Glycerol is a versatile excipient and was found in 864 products listed on the EMC website such as, syrups, creams, capsules, tablets, gels, foams, sprays, injections, mouth wash, etc., and it was listed as an active ingredient in 14 products for treatment of constipation and skin irritation.

Glycerol is accepted as an active ingredient in cough syrups but a search of the literature has not found any published research on the effects of glycerol on cough. This lack of evidence was highlighted in a recent review on the role of glycerol in cough medicines which concluded that “There is, at present, no published research on the efficacy of glycerol as a cough treatment and there is a need for randomized placebo-controlled clinical trials to determine the contribution of this common ingredient in cough medicines to the overall benefit of cough medicines” [12].

In order for a cough medicine to market with a claim to treat cough, it has to be approved and licensed by a health regulatory authority. The cough medicines available in the UK and registered on the emc website have been approved and licensed by authorities such as the UK Medicines and Healthcare Products Regulatory Agency (MHRA) and the European Medicines Agency (EMA). The licensing process will assess the safety of the medicine and the claims for efficacy as a treatment for cough based on the declared active ingredients for the medicine. When considering the approval of medicines containing pharmacological actives such as dextromethorphan or guaifenesin, the health authority will rely on World Health Organization listings of approved medicines, published clinical trials, or in-house clinical trials conducted by the company registering the medicine, and in the case of herbal medicines such as ipecacuanha, licorice, and squill, the health authority will rely on regulations concerning their use as a traditional herbal medicinal products. In the case of excipients such as glycerol and sugars, which are declared as active ingredients in cough medicines, it is not clear what information the health authorities are using to confirm efficacy of the excipient in treating cough as there are no published clinical studies on these excipients.

A sweet syrup formulation is not unique to cough medicines as many infant and children’s analgesic medicines are formulated as sweet viscous syrups containing sweeteners, thickeners, flavors, antimicrobials, and coloring excipients. However, the sweet syrup formulation is to make the medicine more palatable and easy to administer to infants and children, and the formulation does not influence the efficacy of the analgesic medicines, as the adult formulations are nearly always a tablet or capsule containing the analgesic. The difference between these analgesic medicines and cough medicines is that in nearly all cases, the cough medicine is formulated as a syrup rather than a tablet or capsule.

Excipients such as sugars, glycerol, and menthol are now accepted as active ingredients in cough medicines and although there is some clinical support for the efficacy of menthol as an antitussive,, there is no published research on the benefits of sugars and glycerol and more research is needed in this area.

The excipients used in cough medicines are GRAS and often derived from food products and they can be formulated into safe medicines that are popular with consumers and they provide real relief from cough even if the major benefit is a demulcent or placebo effect. The demulcent and placebo effects of cough medicines can be enhanced by the excipients that sweeten, thicken, flavor, and color the medicines and more research is needed in this area to help develop new and more effective medicines.

There is a great need for effective cough medicines and the latest research clinical trials on chronic cough use cough medicines formulated as tablets [30]. It will be important to consider if in the future these medicines are used to treat acute cough if they will be administered to patients as tablets or if pharmaceutical companies will harness the power of excipients to enhance the efficacy of these new medicines. If new cough medicines are to be marketed as syrups rather than tablets, then this may complicate clinical trials on these new medicines, as the placebo effect and other actions of excipients may be the major component of the efficacy of the new cough product [1, 26]. The easiest way forward is to conduct the clinical trials on tablet formulations and to conduct consumer market research on how patients will react to a new cough medicine in tablet form. If the medicine is to be taken regularly for a chronic cough, a tasteless tablet may be the best formulation, as everyday doses of a sweet syrup may be too much for the chronic cough patient.
